# A retrospective audit of an artificial intelligence software for the detection of intracranial haemorrhage used by a teleradiology company in the United Kingdom

**DOI:** 10.1093/bjro/tzae033

**Published:** 2024-10-04

**Authors:** Garry Pettet, Julie West, Dennis Robert, Aneesh Khetani, Shamie Kumar, Satish Golla, Robert Lavis

**Affiliations:** Medica Group Limited, Hastings, TN34 1EA, United Kingdom; Medica Group Limited, Hastings, TN34 1EA, United Kingdom; Qure.ai Technologies Private Limited, Floor 2, Prestige Summit, Halasuru, Bangalore, Karnataka, 560042, India; Qure.ai Technologies Private Limited, United Kingdom; Qure.ai Technologies Private Limited, United Kingdom; Qure.ai Technologies Private Limited, Floor 2, Prestige Summit, Halasuru, Bangalore, Karnataka, 560042, India; Medica Group Limited, Hastings, TN34 1EA, United Kingdom

**Keywords:** artificial intelligence, intracranial haemorrhage, head-ct, teleradiology, real-world medical ai evaluation

## Abstract

**Objectives:**

Artificial intelligence (AI) algorithms have the potential to assist radiologists in the reporting of head computed tomography (CT) scans. We investigated the performance of an AI-based software device used in a large teleradiology practice for intracranial haemorrhage (ICH) detection.

**Methods:**

A randomly selected subset of all non-contrast CT head (NCCTH) scans from patients aged ≥18 years referred for urgent teleradiology reporting from 44 different hospitals within the United Kingdom over a 4-month period was considered for this evaluation. Thirty auditing radiologists evaluated the NCCTH scans and the AI output retrospectively. Agreement between AI and auditing radiologists is reported along with failure analysis.

**Results:**

A total of 1315 NCCTH scans from as many distinct patients (median age, 73 years [IQR 53-84]; 696 [52.9%] females) were evaluated. One hundred twelve (8.5%) scans had ICH. Overall agreement, positive percent agreement, negative percent agreement, and Gwet’s AC1 of AI with radiologists were found to be 93.5% (95% CI, 92.1-94.8), 85.7% (77.8-91.6), 94.3% (92.8-95.5) and 0.92 (0.90-0.94), respectively, in detecting ICH. 9 out of 16 false negative outcomes were due to missed subarachnoid haemorrhages and these were predominantly subtle haemorrhages. The most common reason for false positive results was due to motion artefacts.

**Conclusions:**

AI demonstrated very good agreement with the radiologists in the detection of ICH.

**Advances in knowledge:**

Real-world evaluation of an AI-based CT head interpretation device is reported. Knowledge of scenarios where false negative and false positive results are possible will help reporting radiologists.

## Introduction

Computed tomography (CT) scans are one of the most common radiological investigations performed in hospitals. According to the latest annual statistical release from National Health Service (NHS) England, approximately 7 million CT scans were done in the year 2022/2023 and this represents an increase of about 5.5% from the previous year and more than double the number of scans done in the year 2012/2013.^1^ Among the diverse types of CT scans, non-contrast CT head (NCCTH) is a prominent study due to its established usefulness in the diagnostic workup of patients presenting with acute head injury, polytrauma and suspected stroke as it is non-invasive and has a fast turnaround time.^2^ Because of the marked increase in the number of imaging investigations and the limited expansion in the number of radiologists, the workload of radiologists is continuously on the rise. According to The Royal College of Radiologists (RCR), there is a 29% shortfall of radiologists as of 2022 and this shortfall is predicted to rise to about 40% by 2027.^3^ Usage of artificial intelligence (AI) based solutions and teleradiology are suggested as potential strategies to tackle this increased volume demand and limited reporting supply problem.^4^^,^^5^

AI algorithms are becoming increasingly popular in the field of radiology and many such algorithms are receiving clearance by regulatory bodies including in the subspeciality of neuroimaging.^6^ A recent systematic review of AI algorithms for intracranial haemorrhage (ICH) detection discusses several such potentially useful algorithms but stresses the need for more real-world evidence of their benefits.^7^ As the use of AI is becoming increasingly popular, a deeper understanding of the performance of the AI is important for its optimal use by radiologists. We conducted an evaluation of one such regulatory cleared and commercially available AI-based software device (qER) which has been deployed in a teleradiology practice for assisting in the reporting and prioritization of NCCTH scans by radiologists. The main objective was to evaluate the agreement between AI and radiologists for the presence/absence of ICH.

## Materials and methods

### Ethical considerations

This was a retrospective evaluation from an auditing process already implemented in the teleradiology practice (Medica) as part of standard operating procedures. The Medica Reporting Limited Clinical Governance Committee, Chief Medical Officer, and Caldicott Guardian have approved this evaluation. Only de-identified data was used for statistical analysis.

### About the AI software device and prior evidence

**Figure 1.**
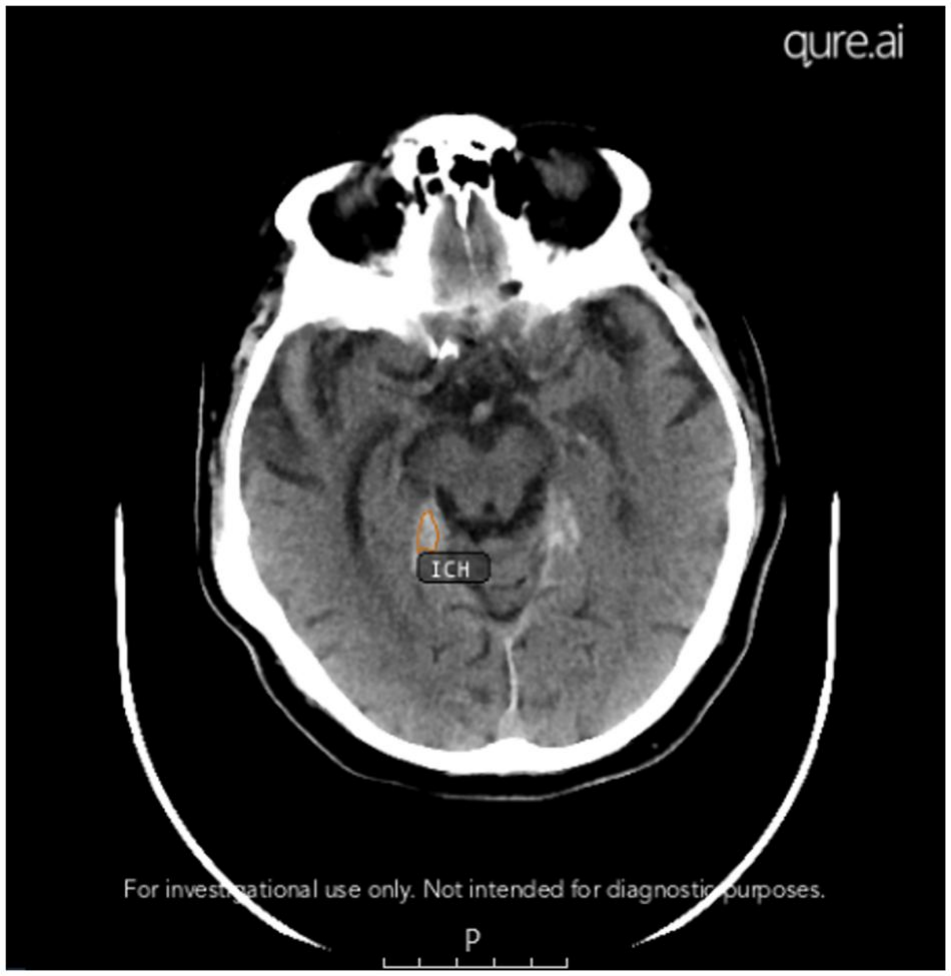
Example of a secondary capture image generated by qER showing a key slice with the presence of ICH.

qER (version 2.0 EU, developed by Qure.ai) is a CE (Conformité Européenne) class IIb and Federal Drug Agency (FDA) 510(k) cleared AI software device for analysing NCCTH scans in patients aged ≥18 years. The device can detect abnormalities such as ICH including its 5 subtypes of extradural (EDH), subdural (SDH), subarachnoid (SAH), intraparenchymal (IPH), and intraventricular (IVH) haemorrhages, mass effect, midline shift, cranial fractures, hypodensities suggestive of infarct and atrophy. The device has regulatory clearance for detecting ICH, mass effect, midline shift, and cranial fractures. The core component of each detection algorithm is a classification convolutional neural network (CNN) that has been trained to detect a specific abnormality.^8^ A scan-level probability score (a value between 0 and 1) for each abnormality is the backend output from the corresponding algorithm. A threshold is applied to the probability score to determine the presence or absence of the target abnormality. A secondary capture (SC) image is generated which informs the radiologist of the presence of any of the target abnormalities if any are present (Figure 1). Scans containing artefacts, post-operative defects, and metal implants are known to cause inaccurate outputs by qER, and these are part of the device warnings so that radiologists are aware of the possibility of inaccurate AI outputs. For this evaluation, we focused on the ICH detection capability of qER as it was deployed in the teleradiology practice for identifying scans with ICH (Figure 1).

In a study conducted in Sweden using data from a stroke registry, qER was found to have approximately 97% sensitivity in detecting non-traumatic ICH, and 95% of the missed ICHs were <1 mm in diameter.^9^ Another study using an external validation dataset of 491 NCCTH scans collected from inpatient and outpatient settings in India, reported a sensitivity of 81.95% (95% CI, 75.99-86.99) in detecting ICH at a high specificity of 90%.^8^ In a study conducted for the purpose of qER regulatory clearance using 1320 NCCTH scans from multiple sites in the United States, the area under the receiver operating characteristic curve (AUC) was reported to be more than 97% for ICH, skull fracture, mass effect, and midline shift.^10^

### Data collection

Medica Group Limited is a telemedicine practice with a UK arm, Medica Reporting Limited, offering teleradiology and telepathology services. The AI device (qER) has been deployed and successfully integrated with the acute teleradiology workflow for assistance in reporting NCCTH scans since the end of 2020. During the real-time reporting of scans, the original reporting radiologists would see a priortization flag in their worklist corresponding to an NCCTH scan if the AI detected the presence of ICH in that scan. The SC image from AI would indicate the presence of ICH, if detected by the AI, along with an overlay around the detected bleed (Figure 1). Information on the subtype of detected ICH was not provided in the SC image.

A subset of all consecutive NCCTH scans that were successfully analysed by the AI device from patients aged ≥18 years referred for emergency and urgent teleradiology reporting from 44 different hospital sites in the United Kingdom during a 4-month period from September 13, 2023 to January 16, 2024 were selected randomly. Scans were identified using an internal audit tool which selected scans for auditing once they have been reported based on simple random sampling.

We determined that a sample of 100-125 scans with radiologists confirmed the presence of ICH would enable us to estimate an anticipated minimum positive percent agreement (PPA) of AI of 85% with radiologists with about 7%-6.25% precision (half-width of the 95% confidence interval [CI]).^11^ Assuming the prevalence of ICH to be about 8% based on knowledge from prior auditing experiences, a sample of approximately 1400 NCCTH scans was desired for this evaluation which would also allow us to estimate an anticipated minimum 90% overall agreement with a higher precision of about 1.58%. The original DICOM (Digital Imaging and Communications in Medicine) NCCTH images, original radiology report, and the SC generated by AI were available in the historical electronic data to be used for this evaluation.

### Auditing process

The original DICOM NCCTH images, original radiology reports, and the AI-generated SC images were retrospectively evaluated by a team of 30 auditing radiologists (5 neuroradiologists and 25 general radiologists) with an average experience of 13 years (median experience of 12 years) in radiological reporting. There was no delineation of cases between neuroradiologists and general radiologists. The auditing radiologists were selected for their consistent high-quality auditing in acute imaging investigations based on prior experience. The general radiologists were all credentialled for CT head reporting. All auditing radiologists were given access to a shared audit worklist, and they randomly selected cases for reviewing from the worklist. The auditor would have been selecting cases based on the clients where they had log-in access to review reports on the radiology information system. All auditing radiologists reviewed the original DICOM scan at first. Then they reviewed the original radiologist report and the AI SC image. Each patient’s data was evaluated by a single auditing radiologist and none of the auditing radiologists evaluated their own previous original radiology report. The images were all reviewed using the Sectra PACS IDS7 (Sectra AB, Sweden) platform. This process of evaluation (auditing) is periodically followed in Medica as part of internal standard operating procedures. The auditing radiologist, after thorough inspection of the original NCCTH image, AI-generated SC, and the original radiology report, was asked to rate each scan using one of the following 5 categories:

- Agree—Great Spot: the auditing radiologist agreed with the positive (presence of ICH) finding by AI and decided this positive finding is a good subtle spot. These are true positive (TP) cases.
- Agree with positive finding: the auditing radiologist agrees with the positive finding by AI. These are also TP cases.
- Agree with negative finding: the auditing radiologist agrees with the negative (absence of ICH) finding by AI. These are true negative (TN) cases.
- Disagree with positive finding: the auditing radiologist disagrees with the positive finding by AI. These are false positive (FP) cases.
- Disagree with negative finding: the auditing radiologist disagrees with the negative finding by AI. These are false negative (FN) cases.

If the auditing radiologist disagreed with the original radiology report, a discrepancy is raised for the report to review the scan as per standard operating procedure in Medica. However, this analysis was out-of-scope for this evaluation in which we only focussed on the auditing outcome (auditing radiologist impressions vs AI). If there was a disagreement with AI, the auditing radiologist also reported the potential reason for disagreement. The auditing radiologist reviewed the scans to scrutinize the AI findings strictly by conducting thorough reviews. A descriptive report of the failure analysis is also reported.

### Statistical analysis

To evaluate the agreement between AI and radiologists, we use the terms overall agreement, PPA, and negative percent agreement (NPA) based on the guidance from FDA^12^ for reporting results from analysis of diagnostic devices when a non-reference standard is used. The calculations of overall agreement, PPA, and NPA are same as that of accuracy, sensitivity, and specificity, respectively, in a typical diagnostic accuracy study.

Point estimates and corresponding 95% exact binomial CI (95% CI) of overall agreement, PPA, NPA, positive predictive value (PPV), and negative predictive value (NPV) are reported. A descriptive analysis of the reasons for false results is also reported for scans where there was disagreement between AI and auditing radiologists. Agreement between the AI tool and the auditing radiologist was also quantified by Gwet’s AC1^13^, Cohen’s kappa and prevalence and bias-adjusted kappa (PABAK).^14^ A multivariable logistic regression model was also fitted with age, sex, and the interaction term between age and sex as independent variables to investigate if there was any association of age and sex with incorrect AI results. The statistical analysis was conducted in R version 4.3.2 (R Foundation for Statistical Computing).

## Results

### Baseline characteristics

**Figure 2.**
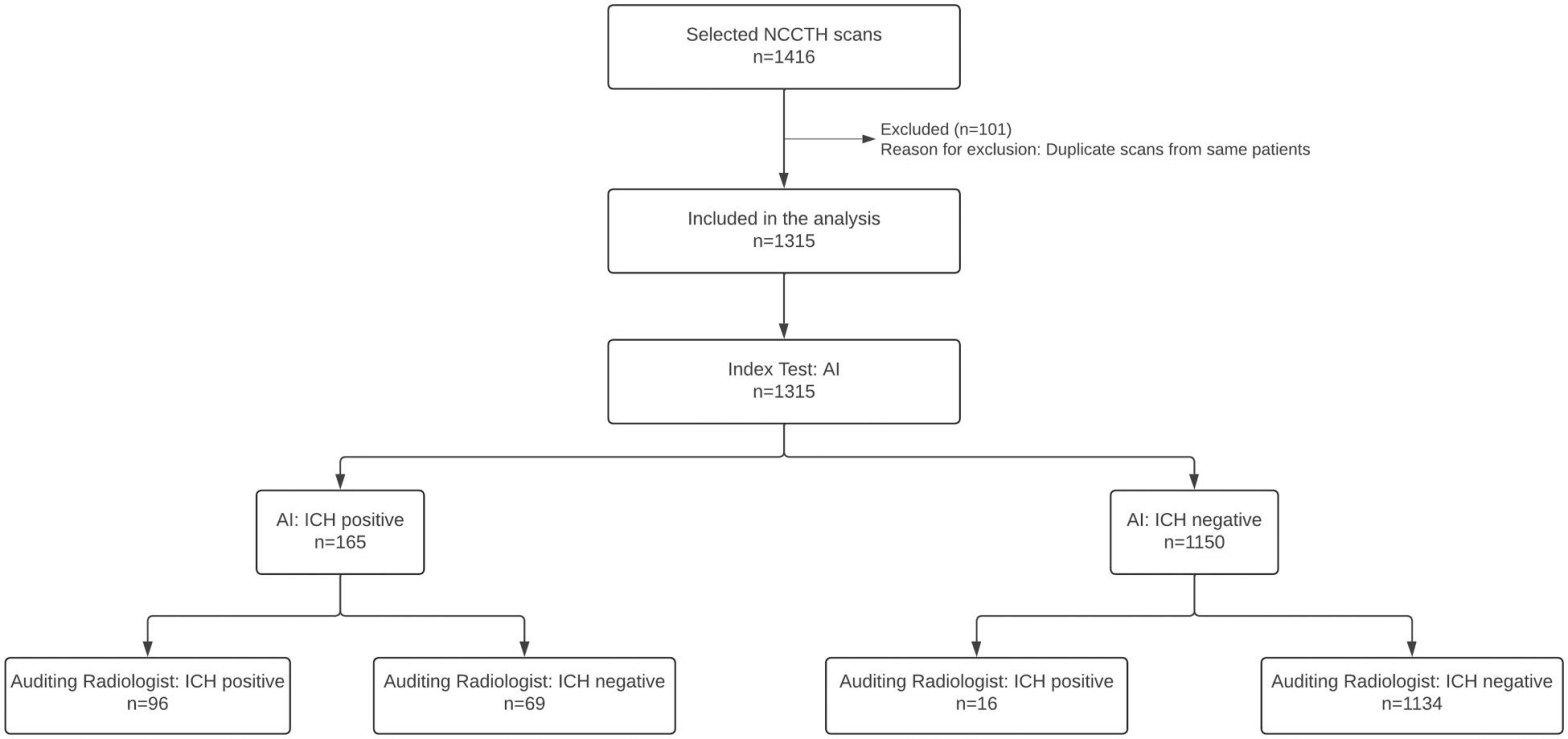
Data flow diagram.

From a potentially eligible pool of 27,432 NCCTH scans, a subset of 1416 scans were randomly sampled from patients aged ≥18 years. These scans were originally also processed by qER in real-time. After excluding 101 duplicate scans, 1315 NCCTH scans from as many distinct patients were available for the final analysis (Figure 2). 619 (47.1%) of the cases were males, 696 (52.9%) were females and the mean age of the patients was 67.7 (median: 73.0, IQR: 53.0-84.0, standard deviation: 20, Table 1). 1256 (95.5%) of the scans were reviewed by the 25 general radiologists and the rest 59 were reviewed by the 5 neuroradiologists. Roughly 90% of the scans were from 25 hospitals and the rest 10% were from the remaining 19 hospitals. The number of scans and basic demographic information of patients from each hospital are detailed in Supplementary Table S1.

**Table 1.** Baseline characteristics.

Baseline characteristic	Overall *n* = 1315	ICH positive *n* = 112	ICH negative *n* = 1203
Age, mean ± SD	68 ± 20	69 ± 16	68 ± 21
Gender			
Female, *n* (%)	696 (53%)	55 (49%)	641 (53%)
Male, *n* (%)	619 (47%)	57 (51%)	562 (47%)

Abbreviations: ICH = intracranial haemorrhage; SD = standard deviation.

Note: Percentages are rounded to the nearest integer.

### Agreement between AI and radiologists

The AI device output was found to be in agreement with the auditing radiologists for the presence and absence of ICH in 93.5% (95% CI, 92.1-94.8) cases. The presence of ICH was confirmed in 112 (8.5%) of the scans as per the auditing radiologist’s interpretation. AI indicated the presence of ICH in 96 of these 112 cases (PPA: 85.7%; 95% CI, 77.8-91.6) and the NPA was found to be 94.3% (95% CI, 92.8-95.5) (Tables 2 and 3). Two (2.1%) of the 96 TP cases were deemed to be “Agree—Great Spot” by the auditing radiologist; one was a subtle SAH, and the other one was a haemorrhage in a surgical cavity in a scan containing motion artefact.

**Table 2.** Contingency table showing ICH classification results.

	Auditing radiologist: ICH	Auditing radiologist: no ICH	Total
AI: ICH	96 (TP)^a^	69 (FP)	165
AI: no ICH	16 (FN)	1134 (TN)	1150
Total	112	1203	1315

Abbreviations: AI = artificial intelligence; FN = false negative cases where auditing radiologist found ICH, but AI finding was negative for ICH; FP = false positive cases where auditing radiologist disagreed with the AI finding positive for ICH; ICH = intracranial haemorrhage; TN = true negative cases where both auditing radiologist and AI agreed for the absence of ICH; TP = true positive cases, where both auditing radiologist and AI agreed for the presence of ICH.

a Two of 96 TP results were deemed as “Agree—Great Spot” by the auditing radiologist.

**Table 3.** Overall results.

Metric	Point estimate	95% CI lower limit	95% CI upper limit
Overall agreement	93.5%	92.1	94.8
PPA	85.7%	77.8	91.6
NPA	94.3%	92.8	95.5
PPV	58.2%	50.3	65.8
NPV	98.6%	97.8	99.2
Gwet’s AC1	0.92	0.90	0.94
PABAK	0.87	0.84	0.90
Cohen’s kappa	0.66	0.59	0.73

Abbreviations: CI = confidence interval; NPA = negative percent agreement; NPV = negative predictive value; PABAK = prevalence and bias-adjusted kappa; PPA = positive percent agreement; PPV = negative predictive value.

An almost perfect agreement is found between the AI device and the auditing radiologist in terms of Gwet’s AC1 (0.92; 95% CI, 0.90-0.94) and PABAK (0.87; 95% CI, 0.84-0.90). The Cohen’s kappa was estimated to be 0.66 (95% CI, 0.59-0.73).

There were no associations of age (*P* = 0.8), sex (*P* = 0.6), or the interaction term of age and sex (*P* = 0.8) with incorrect AI results as per the multivariable logistic regression analysis (Table 4).

**Table 4.** Multivariable logistic regression analysis output.

Independent variables	OR	95% CI	*P*-value
Sex			0.6
Female	1.56	0.36-7.03	
Male			
Age	1.00	0.98-1.01	0.8
Sex × Age	1.00	0.98-1.02	0.8
Male × Age			

Abbreviations: CI = confidence interval; OR = odds ratio.

Note: Dependent variable in the multivariable logistic regression model was a binary variable indicating if there was agreement (value = 1) between the AI and radiologist or not (value = 0); reference category was “1”. The reference category for the independent variable “Sex” was “Female”.

### Failure analysis

There were 16 FN outcomes (FN rate: 14.3%) of which 9 (56.2%), 4 (25.0%), and 3 (18.8%) were due to missed SAH, SDH, and IPH, respectively (Table 5). Of the 9 missed SAH cases, 8 were reported by the auditing radiologist as “small” or “tiny” bleeds. None of the missed ICH cases were reported as significant by auditing radiologists.

**Table 5.** Failure analysis.

Disagreement type	Attributed reason for failure (*n*, %)
False negative (*n* = 16)	Subarachnoid haemorrhage (9, 56.2%)
	Subdural haemorrhage (4, 25%)
	Intraparenchymal haemorrhage (3, 18.8%)
False positive (*n* = 69)	Motion artefacts (21, 30.4%)
	Tumours with acute blood density (15, 21.7%)
	Calcified structures (14, 20.3%)
	Hyperdense venous sinuses (10, 14.5%)
	Aneurysms (2, 2.9%)
	Hyperdense tentorium (1, 1.5%)
	Cerebral oedema (1, 1.5%)
	Unknown (5, 7.2%)

There were 69 FP results indicating a FP rate of 5.7%. 64 (92.7%) of them had a reason for the FP AI outcome documented by the auditing radiologist. Majority of the FP results were due to AI incorrectly flagging bleeds in scans with motion artefacts (*n* = 21, 30.4%) and because AI mistakenly identifying tumours such as meningiomas/haemangiomas (*n* = 15, 21.7%) and calcified structures (*n* = 14, 20.3%) as bleeds (Table 5).

## Discussion

In our analysis, the AI device was found to be in agreement with auditing radiologists for the presence or absence of ICH in 1230 of the 1315 scans as indicated by an overall agreement with auditing radiologists of 93.5% (95% CI, 92.1-94.8). The PPA and NPA were found to be 85.7% (95% CI, 77.8-91.6) and 94.3% (95% CI, 92.8-95.5), respectively, which are slightly higher than what was reported in a prior diagnostic accuracy study of the same AI device conducted using NCCTH scans from in-hospital and outpatient radiology centres in India.^8^ The PPA of the AI device in our evaluation was lower than that reported (approximately 97% sensitivity) in a diagnostic accuracy study conducted using NCCTH scans from patients with spontaneous non-traumatic ICH from a Swedish stroke registry.^9^ It is to be noted that we report agreement metrics such as PPA and NPA between AI and radiologists and hence a one-to-one comparison with typical diagnostic accuracy metrics such as sensitivity and specificity is not possible although the calculations are the same. The differences in the study population characteristics could be another reason for these observed differences. Our study population consisted of NCCTH scans from unfiltered acute cases which were referred for urgent telereporting. In another study conducted in rural India, the sensitivity and specificity of the AI device were reported to be 89% (95% CI, 83-93) and 99% (95% CI, 98-100), respectively, using a sample of consecutive NCCTH scans.^15^

When quantifying the agreement, we observed that compared to Gwet’s AC1 value of 0.92, the Cohen’s kappa estimate was much lower (0.66). This is likely because Cohen’s kappa is known to produce biased results in samples with skewed distribution (prevalence problem).^16^^,^^17^ Gwet’s AC1 is recommended for quantifying agreement in samples with skewed prevalence of target condition.^17^

A systematic review and meta-analysis of various other AI algorithms in detecting ICH are available in the literature.^7^ The reported diagnostic accuracies in those studies are generally comparable with what we have observed in our analysis although a comprehensive comparison is difficult due to variations in the study population and reference standards. Seyam et al reported 93.0% diagnostic accuracy (overall agreement), 87.2% sensitivity (PPA), and 97.8% NPV for another, but similar, AI algorithm.^18^ Gaizo et al studied another similar AI algorithm and reported a sensitivity (PPA) of 75.6%, specificity (NPA) of 92.1%, and accuracy (overall agreement) of 91.7%.^19^ In our analysis, we observed a high NPV of 98.6% (95% CI, 97.8-99.2) for the AI device which might be useful because excluding bleed in a clinical acute stroke case helps in determining the eligibility for thrombolysis.^20^

The strength of our evaluation is that our data reports the agreement of AI in detecting ICH with radiologists in a real-world teleradiology setting. We were also able to report a failure analysis of FP and FN AI results. We found that the AI device tends to miss small volume of SAH. FP results were majorly due to AI flagging calcified structures, tumours, and movement artefacts as ICH. It is to be noted that the device description includes a warning that inaccurate results are likely in scans with movement artefacts and thus radiologists need to be cautious in interpreting AI results in such scenarios. FP (disagreement with positive ICH finding of AI) and FN (disagreement with negative ICH finding of AI) results were not associated with the age and sex of the patients.

Our study is limited by a relatively small number of ICH scans and thus the precision (half-width of the 95% CI) of the point estimate of PPA that we could achieve was about 7%. We also did not have access to the probability scores of AI and thus we could not conduct a receiver-operating-characteristics analysis. Since each scan was audited by only one radiologist, significant inter-reader variability impacting the evaluation cannot be ruled out. We also cannot rule out the possibility of bias due to the unblinded review of auditing radiologists, but this allowed us to report agreement metrics with radiologists, which are good indicators of what can be expected when AI is assisting radiologists, and a failure analysis.

## Conclusion

The AI device demonstrated very good agreement with radiologists in the detection of ICH from NCCTH scans. The optimal use of AI by radiologists can be augmented by knowledge of the scenarios where AI is likely to generate inaccurate outputs. Accurate AI recognition of haemorrhage can enable higher-risk scans to be prioritized for reading thus potentially enabling rapid patient management.

## Supplementary Material

tzae033_Supplementary_Data
