# Supplementary material for: A retrospective audit of an artificial intelligence software for the detection of intracranial haemorrhage used by a teleradiology company in the United Kingdom
Source: BJR Open. 2024 Oct 4;6(1):tzae033. doi: 10.1093/bjro/tzae033 (PMC11522876; doi:10.1093/bjro/tzae033)
Supplement: tzae033_Supplementary_Data [file tzae033_supplementary_data.docx]

**Supplementary Table: Distribution of scans per each hospital**

| **Hospital** | **Number of NCCTH scans (%)**^†^ | **Number of females (%)**^‡^ | **Median age in years**^§^ |
| --- | --- | --- | --- |
| Hospital 1 | 17 (1.3%) | 11 (64.7%) | 76 |
| Hospital 2 | 22 (1.7%) | 8 (36.4%) | 65 |
| Hospital 3 | 27 (2.1%) | 11 (40.7%) | 61 |
| Hospital 4 | 23 (1.7%) | 9 (39.1%) | 79 |
| Hospital 5 | 1 (0.1%) | 0 (0%) | 84 |
| Hospital 6 | 153 (11.6%) | 94 (61.4%) | 65 |
| Hospital 7 | 4 (0.3%) | 4 (100%) | 82 |
| Hospital 8 | 6 (0.5%) | 5 (83.3%) | 68.5 |
| Hospital 9 | 62 (4.7%) | 27 (43.5%) | 75.5 |
| Hospital 10 | 29 (2.2%) | 14 (48.3%) | 81 |
| Hospital 11 | 15 (1.1%) | 4 (26.7%) | 66 |
| Hospital 12 | 34 (2.6%) | 24 (70.6%) | 78.5 |
| Hospital 13 | 52 (4%) | 23 (44.2%) | 78 |
| Hospital 14 | 10 (0.8%) | 6 (60%) | 64.5 |
| Hospital 15 | 1 (0.1%) | 1 (100%) | 79 |
| Hospital 16 | 4 (0.3%) | 4 (100%) | 72 |
| Hospital 17 | 16 (1.2%) | 5 (31.2%) | 74 |
| Hospital 18 | 10 (0.8%) | 6 (60%) | 82.5 |
| Hospital 19 | 27 (2.1%) | 13 (48.1%) | 74 |
| Hospital 20 | 29 (2.2%) | 16 (55.2%) | 73 |
| Hospital 21 | 129 (9.8%) | 71 (55%) | 68 |
| Hospital 22 | 39 (3%) | 22 (56.4%) | 65 |
| Hospital 23 | 12 (0.9%) | 3 (25%) | 84 |
| Hospital 24 | 21 (1.6%) | 14 (66.7%) | 73 |
| Hospital 25 | 15 (1.1%) | 5 (33.3%) | 65 |
| Hospital 26 | 4 (0.3%) | 1 (25%) | 47.5 |
| Hospital 27 | 22 (1.7%) | 12 (54.5%) | 70 |
| Hospital 28 | 1 (0.1%) | 1 (100%) | 30 |
| Hospital 29 | 5 (0.4%) | 2 (40%) | 63 |
| Hospital 30 | 4 (0.3%) | 3 (75%) | 69.5 |
| Hospital 31 | 15 (1.1%) | 4 (26.7%) | 78 |
| Hospital 32 | 9 (0.7%) | 5 (55.6%) | 51 |
| Hospital 33 | 2 (0.2%) | 1 (50%) | 55.5 |
| Hospital 34 | 81 (6.2%) | 44 (54.3%) | 72 |
| Hospital 35 | 59 (4.5%) | 41 (69.5%) | 75 |
| Hospital 36 | 14 (1.1%) | 5 (35.7%) | 77.5 |
| Hospital 37 | 32 (2.4%) | 13 (40.6%) | 79 |
| Hospital 38 | 54 (4.1%) | 28 (51.9%) | 68.5 |
| Hospital 39 | 3 (0.2%) | 2 (66.7%) | 29 |
| Hospital 40 | 28 (2.1%) | 18 (64.3%) | 74.5 |
| Hospital 41 | 145 (11%) | 75 (51.7%) | 78 |
| Hospital 42 | 5 (0.4%) | 3 (60%) | 60 |
| Hospital 43 | 61 (4.6%) | 32 (52.5%) | 79 |
| Hospital 44 | 13 (1%) | 6 (46.2%) | 65 |
| **Total** | **1315 (100%)** | **696 (52.9%)** | **73** |

The last row shows the total n of NCCTH scans, n of females and median age of patients in the overall sample

^†^ Number of non-contrast head CT (NCCTH) scans from each hospital and proportion of contribution

^‡^ Number of female patients from each hospital and proportion in comparison to males

^§^ The median age in years of patients from each hospital
